# The effect of shift work on burnout and occupational fatigue among clinical faculty members during the COVID-19 pandemic

**DOI:** 10.3389/fpubh.2022.973690

**Published:** 2022-10-17

**Authors:** Abdolreza Gilavand

**Affiliations:** Department of Education Development Center, Ahvaz Jundishapur University of Medical Sciences, Ahvaz, Iran

**Keywords:** shift work, burnout, occupational fatigue, clinical faculty members, COVID-19

## Abstract

**Introduction:**

Shift work of clinical faculty members in the COVID-19 pandemic may cause burnout and occupational fatigue and as a result, may reduce the quality of student education and disrupt the treatment of patients, so this study was conducted to evaluate this case.

**Materials and methods:**

The statistical population of this cross-sectional research included all clinical faculty members of Ahvaz Jundishapur University of Medical Sciences in southwestern Iran, who experienced a shift work system (night shift from 8 p.m. to 8 a.m.) during the COVID-19 pandemic, and finally, 71 of them participated in it. The sampling method was also available. Two inventories were used to collect data, namely the Maslach Burnout Inventory [MBI-HSS (MP)] and the Swedish Occupational Fatigue Inventory (SOFI-20).

**Results:**

The self-reported burnout of faculty members was high (mean ± SD = 98.18 ± 17.18), which was graded into the range of emotional exhaustion (38.01 ± 10.2), range of personal accomplishment, (33.75 ± 6.75), and the range of depersonalization (26.42 ± 3.5), respectively. Perceived occupational fatigue of faculty members was also high (M ± SD = 82.25 ± 34.79), which included the dimensions of lack of motivation (18.69 ± 8.65), drowsiness (17.43 ± 8.7), lack of energy (16.33 ± 7.67), physical discomfort (15.65 ± 8.62), and physical stress (13.51 ± 6.9), respectively. In terms of demographic characteristics, occupational fatigue was significantly more common among women.

**Discussion and conclusion:**

The self-reported burnout and occupational fatigue of clinical faculty members due to shift work were reported to be high in this study. Although our knowledge of burnout has advanced in recent years, many gaps in our knowledge still remain. In order for clinical faculty members to properly fulfill their mission to treat patients, educate students, and promote public health, it is necessary to provide all the necessary conditions for their effective activity. Some interventions, such as improving organizational strategies and providing technical solutions, incentives, and occupational facilities, can help reduce or eliminate these problems.

## Introduction

Shift work-related problems are largely chronic. These effects include biological disturbances in physiological processes such as the sleep-wake cycle, physical and physiological health impairment, problems with consciousness, efficiency, safety, and problems for shift workers' family, and social life (1). Performing occupational tasks with little sleep can cause extreme tiredness and drowsiness. This can cause difficulty in concentrating and doing things and can lead to errors and increase the risk of accidents (2). Following the crisis of the COVID-19 pandemic in different parts of the world, the spread of coronavirus in Iran was officially confirmed on 19 February 2020. However, according to some experts, at least a month ago, patients with symptoms of COVID-19 were referred to medical centers that were not identified due to being unknown and a new phenomenon (3). The emergence of the COVID-19 pandemic and its consequences, in addition to the burnout of HCWs, have also caused profound effects on their mental health (4, 5). According to the definition of “Maslach” burnout syndrome is known as a psychological response to work-related stress, which includes three dimensions: the first dimension: emotional exhaustion, which indicates a feeling of pressure and loss of emotional resources in the person. Second dimension: depersonalization makes a person feel negative and indifferent toward patients and those around him. Third dimension: personal accomplishment, which confirms the reduction of a person's sense of competence and negative self-evaluation in relation to doing his work (6). Studies have shown that during the COVID-19 pandemic, physicians and nurses experienced more occupational fatigue and burnout (7, 8). Educating medical students is one of the main tasks of medical universities, and faculty members are important, key, and effective elements in the education process (9). Clinical faculty members are at the heart of the medical community in many ways, because they act as educators, administrators, and—perhaps most importantly—as models for students, assistants, and colleagues. However, these same activities and responsibilities may make them vulnerable to stress and burnout (10). Factors such as burnout and lack of attention to occupational fatigue in universities cause waste of manpower and as a result, inadequate students' training, which will result in the training of inefficient workers. According to some studies, burnout is seen in more than half of physicians, which can be extended to clinical faculty members (11). Physicians who suffer burnout are more likely to make wrong decisions in their profession and may have worse behavior and attitudes toward patients; make more medical mistakes and have a difficult relationship with their colleagues. Burnout among physicians also increases the risk of depression and may increase anxiety, sleep disorders, fatigue, alcohol and drug abuse, marital dysfunction, early retirement, and possibly lead to suicide in serious circumstances (12). Perceived work-related fatigue is an important issue because it can have an adverse effect on employee performance. Also, employee fatigue is one of the main causes of accidents in known work environments. Given the perceived consequences of work-related fatigue, the assessment of fatigue in the workplace is one of the important measures to manage the risk of fatigue (13). In Iran since 1985, the system of providing health services has been integrated into the medical education system, and therefore, in addition to teaching and research in clinical hospitals, clinical faculty members are also engaged in treating patients (14). Because burnout and fatigue due to shift work, especially in the COVID-19 pandemic situation in medical universities, may lead to a decrease in the quality of teaching, lack of motivation in research and indifference to student affairs, and a decrease in flexibility and ability to update what has been learned in the professional world, an increase in medical errors, and a decrease in the ability of classroom management by faculty members, so this study was conducted to evaluate this case. So far, few studies have been conducted to investigate the effects of work shifts in the COVID-19 pandemic conditions on occupational fatigue and burnout of clinical faculty members, and due to the integration of the medical education system and the health care delivery system in Iran, it can be claimed that this is a new and unique study. Considering that shift work of clinical faculty members in the COVID-19 pandemic may cause burnout and occupational fatigue, and as a result, may reduce the quality of student education and disrupt the treatment of patients, so this study was conducted to evaluate this case.

## Materials and methods

The statistical population of this study included all clinical faculty members of Ahvaz Jundishapur University of Medical Sciences in southwestern Iran who experienced a shift work system (night shift from 8 p.m. to 8 a.m.) during the COVID-19 pandemic. This cross-sectional research was conducted for 6 months from 23 October to 21 April 2022 in teaching hospitals affiliated with Ahvaz Jundishapur University of Medical Sciences. Sampling method was also available, in which after identifying the clinical faculty members, an electronic questionnaire was sent to them using virtual social networks such as WhatsApp and Telegram. Inclusion criteria also included being a clinical faculty member in one of the university's departments who had at least 1 year of service in shift work during the COVID-19 pandemic. Initially, all participants completed a written consent form to participate in the research to comply with the research ethics. The data collection method in this study was a questionnaire that in total the demographic information questionnaire, the MBI (Maslach Burnout Inventory), and Swedish Occupational Fatigue Inventory (SOFI-20) were used as follows:

The Persian version of MBI [MBI-HSS (MP)] has been used to assess the burnout of clinical faculty members (13). MBI questionnaire with Cronbach's alpha co-efficient of 0.75 composed of 22 items of self-reported questions. It falls into three main dimensions, emotional exhaustion (EE), depersonalization (DP), and personal accomplishment (PA), with 9, 5, and 8 questions, respectively. Each question expressed the level of burnout with a limited range from zero (never) to six (always). Consistent with previous similar research (14, 15). Mikalauskas et al. (16) reported the internal reliability co-efficient for EE 0.9, DP 0.79, and PA 0.71. How to score the items of this questionnaire is based on the 6-point Likert scale. Items are defined as never, very rarely, rarely, occasionally, frequently, very frequently, and include three dimensions: EE (low 17 and less, average 18–29, high more than 30), DP (low 5 and less, average 6–11, high 12, and more), and PA (low 33 and less, average 34–39, high 40, and more) (17).

Swedish Occupational Fatigue Inventory (SOFI-20) consists of five dimensions: lack of energy, physical stress, physical discomfort, lack of motivation, and drowsiness, and each dimension is measured by four questions. Each question is scored using the 11-point Likert scale from zero (not at all) to 10 (very strongly agree). A fatigue score of up to 33 indicates low fatigue, a score of 34–66 indicates moderate fatigue, and a score of 67 or higher indicates high fatigue (18). The reliability and validity of the Persian version of this questionnaire were assessed using confirmatory factor analysis and Cronbach's alpha co-efficient. The results of confirmatory factor analysis showed that the Persian version of the questionnaire has a good fit. The total Cronbach's alpha co-efficient of the questionnaire was 0.95. Also, Cronbach's alpha co-efficient of different dimensions of the questionnaire was obtained between 0.69 and 0.88 (19). Data were analyzed using SPSS software version 24 at a significant level of 0.05 with the help of mean, standard deviation, percentage, independent *t*-test, Kruskal Wallis and ANOVA. The research project was approved by the Research Ethics Committee of Ahvaz Jundishapur University of Medical Sciences in southwestern Iran (IR.AJUMS.REC.1400.404) http://ethics.research.ac.ir/ProposalCertificateEn.php?id=224841&Print=true&NoPrintHeader=true&NoPrintFooter=true&NoPrintPageBorder=true&LetterPrint=true and in accordance with the ethical guidelines of the 1975 Declaration of Helsinki.

## Results

Table 1 shows the characteristics of the demographic variables of the faculty members. Based on this table, Out of a total of 350 clinical faculty members, 71 of them who were eligible participated in this research.

**Table 1:** Demographic characteristics of faculty members.

	**Frequency (percent)**
**Gender**
Man	42 (59.2)
Woman	29 (40.8)
**Academic rank**
Instructor	2 (2.8)
Assistant professor	54 (76.1)
Associate professor	10 (14.1)
Professor	4 (5.6)
**Age**
31–45	40 (56.3)
46–55	24 (33.8)
>55	7 (9.9)
**Duration of employment as a faculty member**
< 5	23 (32.4)
6–15	34 (47.9)
16–20	5 (7.0)
21–30	9 (12.7)
**Having a clinic=** **doctor's office**
Yes	38 (53.5)
No	33 (46.5)
**Experience of getting Covid-19**
Yes	36 (50.7)
No	35 (49.3)
Total	71 (100.0)

Table 1 shows the distribution of demographic variables of faculty members.

Table 2 shows the descriptive indicators of the main variables of the research based on the average, standard deviation, mean, minimum, and maximum (questionnaires and their various dimensions).

**Table 2:** Descriptive indicators of the main research variables (questionnaires and its various dimensions).

**Demographic characteristics**		**Emotional exhaustion**	**Depersonalization**	**Personal accomplishment**	**Burnout total score**	**Lack of energy**	**Physical tension**	**Physical discomfort**	**Lack of motivation**	**Drowsiness**	**Occupational fatigue total score**
Gender											
	Man	38.64 ± 9.63	26.12 ± 3.15	33.76 ± 6.63	98.52 ± 15.73	14.32 ± 6.33	11.97 ± 5.54	14.46 ± 8.88	16.51 ± 7.81	15.54 ± 7.37	72.8 ± 31.42
	Woman	37.1 ± 11.09	26.86 ± 3.98	33.72 ± 7.03	97.69 ± 19.37	19.17 ± 8.56	15.69 ± 8.07	17.39 ± 8.05	21.76 ± 8.96	20.21 ± 9.84	96.07 ± 35.37
	*P*-value	0.622 (0.536)	−0.877 (0.384)	0.023 (0.982)	0.2 (0.842)	**−2.729 (0.008)**	**−2.284 (0.025)**	−1.397 (0.167)	**−2.603 (0.011)**	**−2.257 (0.027)**	**−2.869 (0.005)**
Academic rank											
	Instructor	28.5 ± 13.43	29.5 ± 0.71	36 ± 4.24	94 ± 18.38	30.5 ± 2.12	27.5 ± 0.71	30.5 ± 3.54	34 ± 8.48	32.5 ± 6.36	155 ± 15.56
	Assistant professor	38.91 ± 9.3	26.33 ± 3.64	33.55 ± 6.54	98.79 ± 16.11	15.23 ± 7.68	12.68 ± 6.77	14.44 ± 7.94	17.92 ± 8.49	15.88 ± 7.89	76.94 ± 32.65
	Associate professor	37.3 ± 11.48	26.8 ± 2.39	35.4 ± 6.67	99.5 ± 18.67	17.7 ± 6.49	14.6 ± 6.57	15.3 ± 7.42	18.1 ± 7.81	18.8 ± 7.87	84.5 ± 30.79
	Professor	37.75 ± 14.88	27.25 ± 1.71	35.5 ± 6.76	100.5 ± 19.26	19 ± 4.24	14 ± 4.55	23 ± 13.39	21.5 ± 8.88	26.25 ± 12.39	103.75 ± 39.68
	*P*-value	1.568 (0.667)	2.109 (0.55)	1.426 (0.699)	0.362 (0.948)	6.385 (0.094)	6.191 (0.103)	6.233 (0.101)	4.778 (0.189)	**8.2 (0.042)**	7.651 (0.054)
Age											
	31–45	36.17 ± 9.26	26.42 ± 3.84	33.05 ± 7.17	95.65 ± 17.15	16.5 ± 7.36	13.35 ± 7.44	16.2 ± 8.65	20.15 ± 9.01	17.92 ± 8.78	84.12 ± 35.81
	46–55	42.08 ± 9.66	26.42 ± 3.33	34.62 ± 5.54	103.12 ± 15.31	14.95 ± 8.34	13.3 ± 5.88	13.09 ± 6.54	15.69 ± 7.09	14.5 ± 6.45	73.32 ± 28.23
	>55	34.57 ± 14.05	26.43 ± 2.15	34.71 ± 8.44	95.71 ± 22.06	19.86 ± 6.82	15.14 ± 7.62	20.57 ± 12.29	20.14 ± 9.79	23.85 ± 11.42	99.57 ± 43.85
	*P*-value	5.811 (0.055)	0.424 (0.809)	0.95 (0.622)	3.045 (0.218)	1.993 (0.369)	0.48 (0.787)	2.732 (0.255)	3.669 (0.16)	4.592 (0.101)	2.518 (0.284)
Duration of employment as a faculty member											
	< 5	37.04 ± 8.56	26.65 ± 3.19	32.65 ± 5.92	96.35 ± 14.33	16.39 ± 8.22	13.39 ± 7.7	14.56 ± 8.67	18.78 ± 9.59	16.13 ± 8.57	79.26 ± 38.49
	6–15	38.06 ± 10.41	26.38 ± 4.13	33.94 ± 7.41	98.38 ± 18.84	16.33 ± 7.39	13.39 ± 6.58	15.73 ± 8.19	18.97 ± 8.05	17.15 ± 8.35	81.57 ± 32.26
	16–20	36 ± 15.57	24 ± 1	31.4 ± 6.77	91.4 ± 21.96	14.8 ± 12.58	14.2 ± 10.35	16.5 ± 9.68	16.6 ± 11.63	19 ± 11.13	91.25 ± 49.24
	21–30	41.44 ± 11.04	27.33 ± 1.94	37.11 ± 5.71	105.89 ± 14.41	17 ± 4.58	13.89 ± 4.48	17.78 ± 10.57	18.55 ± 7.89	21.11 ± 9.72	88.33 ± 32.09
	*P*-value	1.766 (0.622)	5.13 (0.163)	4.502 (0.212)	3.057 (0.383)	0.535 (0.911)	0.346 (0.951)	0.827 (0.843)	0.232 (0.972)	2.13 (0.546)	0.824 (0.844)
Having a clinic= doctor's office											
	Yes	37.63 ± 10.75	26.26 ± 3.19	34.18 ± 6.82	98.08 ± 17.25	16.53 ± 7.72	12.78 ± 6.24	15.36 ± 8.71	18.58 ± 8.59	17.71 ± 9.48	80.97 ± 34.89
	No	38.45 ± 9.67	26.61 ± 3.87	33.24 ± 6.74	98.3 ± 17.37	16.09 ± 7.71	14.37 ± 7.62	16 ± 8.62	18.81 ± 8.84	17.09 ± 7.77	83.81 ± 35.17
	*P*-value	−0.337 (0.737)	−0.409 (0.684)	0.584 (0.561)	−0.054 (0.957)	0.234 (0.816)	−0.957 (0.342)	−0.301 (0.764)	−0.112 (0.911)	0.289 (0.773)	−0.334 (0.739)
Experience of getting Covid-19											
	No	38.39 ± 10.87	26.52 ± 3.43	34.05 ± 6.55	98.97 ± 16.54	15.34 ± 7.64	12.37 ± 6.32	15.76 ± 9.45	19.34 ± 9.91	17.41 ± 8.95	81.47 ± 36.34
	Yes	37.62 ± 9.61	26.31 ± 3.62	33.42 ± 7.02	97.37 ± 18.03	17.31 ± 7.67	14.66 ± 7.35	15.54 ± 7.86	18.03 ± 7.25	17.46 ± 8.59	83 ± 33.72
	*P*-value	0.312 (0.756)	0.255 (0.8)	0.389 (0.698)	0.39 (0.698)	−1.077 (0.285)	−1.395 (0.168)	0.106 (0.916)	0.633 (0.529)	−0.021 (0.983)	−0.181 (0.857)
	Total	38.01 ± 10.2	26.42 ± 3.5	33.75 ± 6.75	98.18 ± 17.18	16.33 ± 7.67	13.51 ± 6.9	15.65 ± 8.62	18.69 ± 8.65	17.43 ± 8.7	82.25 ± 34.79

The bold values indicates significance of the data. P ≥ 0.005.

The results of the study of burnout range showed that the ranges of emotional exhaustion and depersonalization are high. But the range of personal accomplishments is moderate.

The self-reported burnout of faculty members was high (mean ± SD = 98.18 ± 17.18), which was graded into the range of EE (38.01 ± 10.2), range of PA (33.75 ± 6.75), and the range of DP (26.42 ± 3.5), respectively.

Perceived occupational fatigue of faculty members was also high (M ± SD = 82.25 ± 34.79), which included the dimensions of lack of motivation (18.69 ± 8.65), drowsiness (17.43 ± 8.7), lack of energy (16.33 ± 7.67), physical discomfort (15.65 ± 8.62), and physical stress (13.51 ± 6.9), respectively.

Studying the relationship between the main variables of the study and demographic characteristics using an independent *t*-test showed that the gender factor is significant in the total score of occupational fatigue (*P* = 0.005) and women perceive more fatigue. Accordingly, and based on the dimensions of occupational fatigue, the dimensions of lack of energy (*P* = 0.008), physical stress (*P* = 0.025), lack of motivation (*P* = 0.011), and drowsiness (*P* = 0.027) were significant, but in the dimension of physical discomfort (*P* = 0.167) was not significant.

Studying the relationship between the main variables of the research and the academic rank of faculty members, using the Kruskal–Wallis test, showed that the factor was only significant in drowsiness (*P* = 0.042) of the ranges of occupational fatigue (and instructors reported more fatigue). But in other ranges, the factor was not significant.

Studying the relationship of the main variables of the research with age and duration of employment as a clinical faculty member using Kruskal–Wallis test showed that it is not significant. Also, the study of the relationship between the main variables of the research with the factors of employment mode and experience of getting COVID-19 using independent *t*-test was not significant.

**Figure 1:**
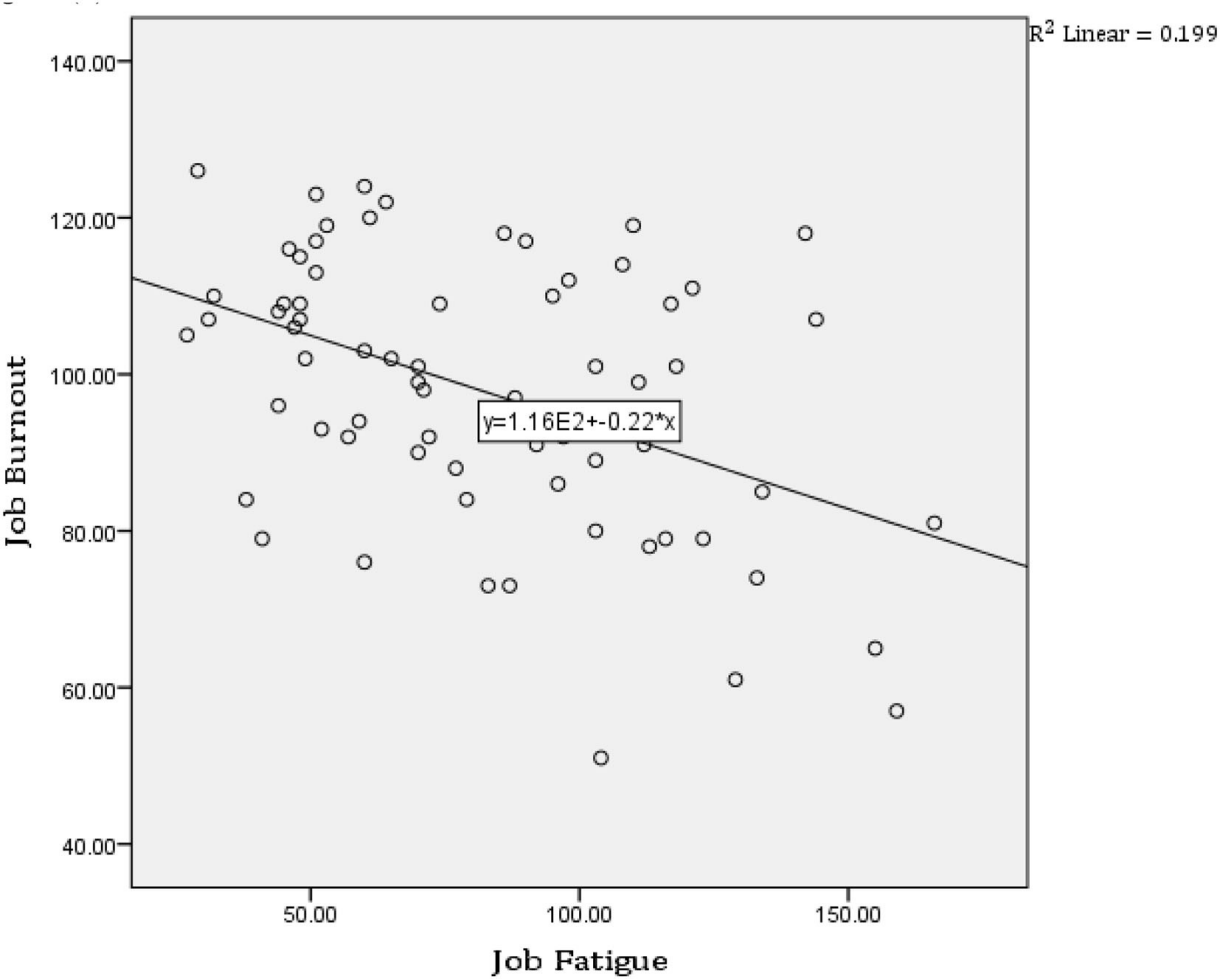
Evaluation of the relationship between the dimensions of the burnout inventory and the dimensions of the occupational fatigue inventory.

Table 3 and Figure 1 show the results of the correlation co-efficient test that examines the relationship between the dimensions of the burnout inventory and the dimensions of the occupational fatigue inventory and the general results showed that there is a relationship between clinical faculty members' burnout and occupational fatigue.

**Table 3:** Evaluation of the relationship between the dimensions of the burnout inventory and the dimensions of the occupational fatigue inventory.

		**Emotional exhaustion**	**Depersonalization**	**Personal accomplishment**	**Burnout total score**
Lack of energy	Correlation co-efficient	−0.533	−0.218	−0.208	−0.443
	*P*-value	< 0.001	0.069	0.083	< 0.001
Physical tension	Correlation co-efficient	−0.424	−0.148	−0.191	−0.357
	*P*-value	< 0.001	0.220	0.113	0.002
Physical discomfort	Correlation co-efficient	−0.480	−0.325	−0.266	−0.454
	*P*-value	< 0.001	0.006	0.027	< 0.001
Lack of motivation	Correlation co-efficient	−0.416	−0.181	−0.120	−0.331
	*P*-value	< 0.001	0.134	0.324	0.005
Drowsiness	Correlation co-efficient	−0.466	−0.243	−0.090	−0.359
	*P*-value	< 0.001	0.044	0.462	0.002
Occupational fatigue total score	Correlation co-efficient	−0.524	−0.272	−0.208	−0.446
	*P*-value	< 0.001	0.024	0.087	< 0.001

## Discussion and conclusion

Based on the results of this study, the overall average score of self-reported burnout and occupational fatigue of clinical faculty members due to shift work were reported to be high during the COVID-19 pandemic. In other words, work shifts during the COVID-19 pandemic may have increased occupational fatigue and burnout of clinical faculty members.

Bahmani conducted a study entitled “the effect of work shifts in the Covid-19 pandemic condition on burnout in nurses with a mediating role of Covid-19 stress,” and concluded that shift work-related disorders have a significant effect on burnout and stress of getting COVID-19 in nurses. In this study, the stress of COVID-19 on nurses' burnout has also reported to be significant (3). Tan et al. (20) surveyed burnout and related factors among Health Care Workers (HCWs) in Singapore during COVID-19 pandemic, in which 3,075 people participated. In this study, a high level of burnout was reported, and employees who had the shifts longer than 8 h experienced more burnout. Sharifi et al. (21) conducted a meta-analysis study to investigate the burnout status of HealthCare Professionals (HCPs) involved in the COVID-19 pandemic and after reviewing 12 studies around the world, they concluded that by paying attention to employees' mental health issues, reducing workload by adjusting their shift work, reducing job-related stressors and creating a healthy work environment, it may be possible to prevent or reduce burnout. In a systematic review study, Lluch et al. (22) reviewed 76 articles examining the effect of the COVID-19 epidemic on burnout and occupational fatigue in healthcare personnel, which have reported high levels. Alsulimani et al. (23) conducted a study to investigate the burnout of healthcare workers during the COVID-19 pandemic in Saudi Arabia. In this study, the rate of burnout was reported to be 75%. In a study, Morgantini et al. examined the burnout of Healthcare Professionals (HCPs) involved in COVID-19 pandemic. In this study, 2,707 people from 60 different countries were evaluated, 51% of whom reported burnout. In this study, burnout was mostly due to high workload, job stress, and time pressure and limited organizational support (24). In a burnout study, Kase et al. (7) examined US pediatric physicians before and after the COVID-19 pandemic, and reported significant differences. In this study, cited as the first comparison of burnout scores before and after the pandemic in a national pediatric subgroup, nearly 40 percent of respondents felt that their contribution to the COVID-19 pandemic was underestimated by their institutions (7). In a study, Akram et al. (25) reported very high levels of burnout among Pakistani clinical faculty members from July 2018 to February 2019. In a study to examine the burnout of physicians working at a hospital in São Paulo, Brazil, in the COVID-19 pandemic, Fumis et al. reported it as high (26).

In this study, women's occupational fatigue was reported to be higher than men's. In addition to their jobs, Iranian women are also responsible for housework, child care, etc. Also, in the workplace, they have to observe the Islamic veil, which can increase their occupational fatigue. A meta-analysis review reviewed 28 published articles and concluded that female HCWs are at increased risk of stress, burnout, and depression during the COVID-19 pandemic (27). Studies show that female physicians are more likely to face sexism, gender biases, delayed personal life decisions, and barriers to professional advancement, all of which may contribute to burnout and occupational fatigue (28).

This study highlights the importance of addressing burnout among healthcare workers.

## Conclusion

The self-reported burnout and occupational fatigue of clinical faculty members due to shift work were reported to be high in this study. Although our knowledge of burnout has advanced in recent years, many gaps in our knowledge still remain. In order for clinical faculty members to properly fulfill their mission to treat patients, educate students, and promote public health, it is necessary to provide all the necessary conditions for their effective activity. Some interventions, such as improving organizational strategies and providing technical solutions, incentives, and occupational facilities, can help reduce or eliminate these problems.

### Limitations

This is a cross sectional study and the reverse bias in the causal relationship is possible. For the correlation between burnout and independent variables, we used the sum of burnout scores and not a logistic model with the prevalence of burnout expressed in frequency and percentage of burnout (as a combination of high EE, high DP and low PA) in our sample (29). Also, although two standard questionnaires were used in this study to measure burnout and occupational fatigue based on the self-report of clinical faculty members, there are other possible factors that can contribute to burnout and occupational fatigue that were not included in our study. Future studies are advised to use qualitative methods such as interviews to reduce this limitation.

## Data availability statement

The original contributions presented in the study are included in the article/supplementary material, further inquiries can be directed to the corresponding author/s.

## Ethics statement

This research has been carried out in line with the research plan No. U-00187 approved by Ahvaz Jundishapur University of Medical Sciences. Written informed consent was obtained from all participants for their participation in this study.

## Author contributions

AG designed research, conducted research, analyzed data, wrote manuscript, had primary responsibility for final content, and read and approved the final manuscript.

## Conflict of interest

The author declares that the research was conducted in the absence of any commercial or financial relationships that could be construed as a potential conflict of interest.

## Publisher's note

All claims expressed in this article are solely those of the authors and do not necessarily represent those of their affiliated organizations, or those of the publisher, the editors and the reviewers. Any product that may be evaluated in this article, or claim that may be made by its manufacturer, is not guaranteed or endorsed by the publisher.
